# CSF CXCL10, CXCL9, and Neopterin as Candidate Prognostic Biomarkers for HTLV-1-Associated Myelopathy/Tropical Spastic Paraparesis

**DOI:** 10.1371/journal.pntd.0002479

**Published:** 2013-10-10

**Authors:** Tomoo Sato, Ariella Coler-Reilly, Atae Utsunomiya, Natsumi Araya, Naoko Yagishita, Hitoshi Ando, Junji Yamauchi, Eisuke Inoue, Takahiko Ueno, Yasuhiro Hasegawa, Kusuki Nishioka, Toshihiro Nakajima, Steven Jacobson, Shuji Izumo, Yoshihisa Yamano

**Affiliations:** 1 Department of Rare Diseases Research, Institute of Medical Science, St. Marianna University School of Medicine, Kawasaki, Kanagawa, Japan; 2 Department of Hematology, Imamura Bun-in Hospital, Kagoshima, Japan; 3 Department of Biostatistics, School of Pharmacy, Kitasato University, Tokyo, Japan; 4 Unit of Medical Statistics, St. Marianna University School of Medicine, Kawasaki, Kanagawa, Japan; 5 Division of Neurology, Department of Internal Medicine, St. Marianna University School of Medicine, Kawasaki, Kanagawa, Japan; 6 Institute of Medical Science, Tokyo Medical University, Tokyo, Japan; 7 Department of Biomedical Engineering, Osaka Institute of Technology, Osaka, Japan; 8 Viral Immunology Section, Neuroimmunology Branch, National Institutes of Health, Bethesda, Maryland, United States of America; 9 Molecular Pathology, Center for Chronic Viral Diseases, Kagoshima University, Kagoshima, Japan; University of Washington, United States of America

## Abstract

**Background:**

Human T-lymphotropic virus type 1 (HTLV-1) -associated myelopathy/tropical spastic paraparesis (HAM/TSP) is a rare chronic neuroinflammatory disease. Since the disease course of HAM/TSP varies among patients, there is a dire need for biomarkers capable of predicting the rate of disease progression. However, there have been no studies to date that have compared the prognostic values of multiple potential biomarkers for HAM/TSP.

**Methodology/Principal Findings:**

Peripheral blood and cerebrospinal fluid (CSF) samples from HAM/TSP patients and HTLV-1-infected control subjects were obtained and tested retrospectively for several potential biomarkers, including chemokines and other cytokines, and nine optimal candidates were selected based on receiver operating characteristic (ROC) analysis. Next, we evaluated the relationship between these candidates and the rate of disease progression in HAM/TSP patients, beginning with a first cohort of 30 patients (Training Set) and proceeding to a second cohort of 23 patients (Test Set). We defined “deteriorating HAM/TSP” as distinctly worsening function (≥3 grades on Osame's Motor Disability Score (OMDS)) over four years and “stable HAM/TSP” as unchanged or only slightly worsened function (1 grade on OMDS) over four years, and we compared the levels of the candidate biomarkers in patients divided into these two groups. The CSF levels of chemokine (C-X-C motif) ligand 10 (CXCL10), CXCL9, and neopterin were well-correlated with disease progression, better even than HTLV-1 proviral load in PBMCs. Importantly, these results were validated using the Test Set.

**Conclusions/Significance:**

As the CSF levels of CXCL10, CXCL9, and neopterin were the most strongly correlated with rate of disease progression, they represent the most viable candidates for HAM/TSP prognostic biomarkers. The identification of effective prognostic biomarkers could lead to earlier detection of high-risk patients, more patient-specific treatment options, and more productive clinical trials.

## Author Summary

HTLV-1-associated myelopathy/tropical spastic paraparesis (HAM/TSP) is a rare neurodegenerative disease caused by infection with human T-lymphotropic virus type 1 (HTLV-1). HTLV-1 infects 10–20 million people worldwide, and, depending on the region, 0.25–3.8% of infected individuals develop HAM/TSP. As the disease progresses, chronic inflammation damages the spinal cord and lower limb and bladder function gradually decline. In the worst cases, even middle-aged patients can become perpetually bedridden. Today, there are treatments that may alleviate the symptoms to a certain degree, but there is no cure that can halt disease progression, and there are no known biomarkers to indicate the level and speed of disease progression. In this study, we successfully identified three promising candidate biomarkers. We believe that the use of these biomarkers could lead to more accurate prognoses and more prudent, patient-specific treatment plans. We not only hope that these biomarkers are sensitive enough to use as selection criteria for clinical trials, but also that measurements of these biomarkers can be used to accurately evaluate drug effectiveness. In short, the biomarkers we identified have the potential to help more effectively treat current HAM/TSP patients and to pave the way for new drugs to potentially cure future HAM/TSP patients.

## Introduction

Human T-lymphotropic virus type 1 (HTLV-1) is a human retrovirus associated with persistent infection of T-cells [1]. While the majority of HTLV-1-infected individuals remain asymptomatic, approximately 2.5–5% develop an aggressive T-cell malignancy, termed adult T-cell leukemia (ATL) [2], [3] and 0.3–3.8% develop a serious chronic neuroinflammatory disease, termed HTLV-1-associated myelopathy/tropical spastic paraparesis (HAM/TSP) [4]–[6]. Aside from Japan, endemic areas for this virus and the associated disorders are mostly located in developing countries in the Caribbean, South America, Africa, the Middle East, and Melanesia [7], [8], which may explain why these conditions have remained ill-defined and virtually untreatable for so long [9].

HAM/TSP is characterized by unremitting myelopathic symptoms such as spastic paraparesis, lower limb sensory disturbance, and bladder/bowel dysfunction [10], [11]. Although the symptoms of HAM/TSP have been well documented for quite some time, the rate at which these symptoms progress has only recently become a point of interest. The clinical course of HAM/TSP has classically been described very simply as insidious onset and continuous progression [12], but recent reports have hinted at a more complex, heterogeneous pool of patients with differing clinical needs. Recent studies have shown that although HAM/TSP usually progresses slowly and without remission as per the classical description, there is a subgroup of patients whose conditions decline unusually quickly and who may be unable to walk within two years of onset and another subgroup whose conditions decline unusually slowly and who may only display very mild symptoms [13]–[15]. It is only logical that these patients should receive treatments tailored to suit their individual needs rather than identically aggressive treatments. Unfortunately, clinicians are currently only able to distinguish between these different groups by observing the way a patient's disease progresses over time, usually years; clinicians often decide to treat the patients immediately and identically rather than wait and allow the disease to progress further. Therein lies the dire need for biomarkers with the power to forecast the rate and extent of disease progression and enable clinicians to make more accurate prognoses and prescribe the most appropriate and effective treatments in a timely manner.

Several candidate prognostic biomarkers with elevated levels in HAM/TSP patients have already been identified in the peripheral blood and cerebrospinal fluid (CSF). In the peripheral blood, such candidates include the HTLV-1 proviral load in peripheral blood mononuclear cells (PBMCs) and serum levels of the soluble IL-2 receptor (sIL-2R) [16], [17]. The level of neopterin in the CSF has been reported to be a useful parameter for detecting cell-mediated immune responses in the spinal cord of HAM/TSP patients and the CSF anti-HTLV-1 antibody titer has been shown to be associated both with CSF neopterin levels and the severity of clinical symptoms [18]–[20]. In addition, several cytokines have been detected in the CSF and/or spinal cord of HAM/TSP patients, including interleukin (IL)-1β, granulocyte-macrophage colony-stimulating factor (GM-CSF), interferon (IFN)-γ, and tumor necrosis factor (TNF)-α [21]–[24]. Some chemokines, such as chemokine (C-X-C motif) ligand (CXCL) 9, CXCL10, and chemokine (C-C motif) ligand (CCL) 5, have been shown to be substantially elevated in both the blood and the CSF with respect to asymptomatic carriers (ACs) or patients with other neurological diseases such as multiple sclerosis [25]–[28]. This is the first study to compare the adequacies of several of these candidate biomarkers for forecasting the rate of disease progression.

We hypothesized the existence of biomarkers capable of differentiating stable and deteriorating HAM/TSP patients. In this retrospective study, a preliminary experiment was first conducted to select the most promising candidate biomarkers by comparing blood and CSF levels in HAM/TSP patients and control subjects (Figure S1). Four candidate blood markers (sIL-2R, CXCL9, CXCL10, and proviral load) and five candidate CSF markers (CXCL9, CXCL10, neopterin, cell count, and anti-HTLV-1 antibody titer) were selected. To evaluate the relative effectiveness of these candidate biomarkers for predicting rate of disease progression, a classification system was created and HAM/TSP patients were designated as either deteriorating or relatively stable. The levels of candidate biomarkers were then compared between the two patient groups. In the current study, we identified three viable candidates for HAM/TSP prognostic biomarkers that could lead to more accurate prognoses and more prudent, patient-specific treatment plans.

## Materials and Methods

### Ethical considerations

The study was designed and conducted in accordance with the tenets of the Declaration of Helsinki. The protocol in this study was approved by the Ethics Review Committee of St. Marianna University School of Medicine (No. 1646). Prior to the collection of blood or CSF samples, all subjects gave written informed consent permitting the analysis of their samples for research purposes as part of their clinical care.

### Subjects

Between April 2007 and February 2013, we enrolled 53 HAM/TSP patients according to the inclusion and exclusion criteria shown in Table 1, and divided them into two cohorts based on the chronological order of their doctor's visits: a 30-patient Training set and a 23-patient Test set. Demographics and clinical characteristics of the Training set and Test set are shown in Table 2 and Table 3, respectively. Between April 2007 and December 2009, we enrolled 22 HTLV-1-infected ACs as control subjects for blood analysis and eight HTLV-1-infected subjects (seven ACs, one patient with smoldering ATL) as control subjects for CSF analysis according to the inclusion and exclusion criteria shown in Table 1. These two groups were not mutually exclusive; some ACs donated both blood and CSF to this study. Demographics of control subjects as compared to the HAM/TSP patients are shown in Table S1.

**Table 1:** Inclusion and exclusion criteria for this study.

	HAM/TSP	Control for Blood	Control for CSF
**Inclusion Criteria**	Willing and able to give informed consent		
	HTLV-1 seropositive individuals conformed by CLEIA and Western blot		
	Diagnosed with HAM/TSP as defined by WHO criteria		Choose to provide CSF for the purposes of differential diagnosis
**Exclusion Criteria**	History of treatment with corticosteroids or other immunomodulating drugs (interferon, cyclosporin, methotrexate, etc.)		
	Diagnosed with an autoimmune disease or other chronic inflammatory disorder aside from HAM/TSP		
	Diagnosed with additional disease affecting gait disturbance (e.g. parkinsonism, rheumatoid arthritis, cervical spondylosis, brain infarction, etc.)		
	History of severe urinary infection, decubitus scars, pneumonia, deep venous thrombosis, or other condition potentially affecting disease course within the last four years	Diagnosed with HAM/TSP as defined by WHO criteria	
	Diagnosed with adult T-cell leukemia (ATL)		

CLEIA = chemiluminescent enzyme immunoassay.

**Table 2:** Demographics and clinical characteristics of HAM/TSP patients (Training Set).

	Total	Stable HAM/TSP	Deteriorating HAM/TSP	
	n = 30	n = 14	n = 11	*p*-value*
**Demographics**				
Age, y**	58 [37–75]	54.5 [39–75]	62 [53–72]	0.0183†
Female sex	80.0%	64.3%	90.9%	0.1696‡
**Clinical characteristics**				
Age of onset, y**	48 [20–70]	33 [20–58]	57 [40–70]	0.0021†
Disease duration, y**	12.5 [1–33]	19 [7–33]	9 [1–13]	0.0021†
OMDS**	6 [2–11]	5 [2–9]	8 [5–11]	0.0065†

In the Training set, deteriorating patients were significantly older, experienced disease onset later in life, had been living with the disease for shorter periods, and were more severely disabled (OMDS).

* Stable HAM/TSP vs Deteriorating HAM/TSP.

** Data are expressed as median [range].

† By Mann-Whitney test.

‡ By Fisher's exact test.

OMDS = Osame's Motor Disability Score.

**Table 3:** Demographics and clinical characteristics of HAM/TSP patients (Test Set).

	Total	Stable HAM/TSP	Deteriorating HAM/TSP	
	n = 23	n = 11	n = 9	*p*-value*
**Demographics**				
Age, y**	58 [22–75]	61 [22–75]	59 [48–68]	0.8491†
Female sex	78.3%	81.8%	77.8%	1.000‡
**Clinical characteristics**				
Age of onset, y**	43 [12–70]	40 [14–70]	51 [39–63]	0.0184†
Disease duration, y**	9 [2–41]	19 [5–41]	6 [2–14]	0.0148†
OMDS**	5 [2–8]	5 [4–8]	5 [4–8]	0.4526†

In the Test set, deteriorating patients experienced disease onset later in life and had been living with the disease for shorter periods, but there were no significant differences in current age or OMDS.

* Stable HAM/TSP vs Deteriorating HAM/TSP.

** Data are expressed as median [range].

† By Mann-Whitney test.

‡ By Fisher's exact test.

OMDS = Osame's Motor Disability Score.

### Sample preparation

Blood and/or CSF samples were obtained within a one-hour window for each subject. Peripheral blood samples were collected in heparin-containing blood collection tubes and serum-separating tubes. Plasma and PBMCs were obtained from the former tubes and serum was obtained from the latter. PBMCs were isolated with standard procedures using Pancoll® density gradient centrifugation (density 1.077 g/mL; PAN-Biotech GmbH, Aidenbach, Germany). Plasma and serum samples were stored at −80°C until use. CSF was collected in polypropylene tubes. A small amount of CSF was used for routine laboratory tests, which included total protein, cell count, and IgG level. The remaining CSF was aliquoted into cryotubes and stored at −80°C until undergoing further analysis. All tests in this study were performed on samples from these frozen stocks.

### Measurement of blood candidate markers

The serum concentration of sIL-2R was determined using an ELISA (Cell Free N IL-2R; Kyowa Medex Ltd., Tokyo, Japan). HTLV-1 proviral load was measured using real-time PCR, following DNA extraction from PBMCs, as previously described [29]–[31]. Plasma levels of IL-1β, TNF-α, and IFN-γ were measured using a cytometric bead array (CBA) (BD Biosciences, Franklin Lakes, NJ USA), which was used according to the manufacturer's instructions. Plasma concentrations of CXCL9, CXCL10, CXCL11, and CCL5 were also measured using a CBA (BD Biosciences).

### Measurement of CSF candidate markers

CSF cell count was determined using the Fuchs–Rosenthal chamber (Hausser Scientific Company, Horsham PA USA). Total protein and IgG levels in the CSF were measured using a pyrogallol red assay and a turbidimetric immunoassay, respectively. The anti-HTLV-1 antibody titer was determined using the gelatin particle agglutination test (Serodia-HTLV-1; Fujirebio, Tokyo, Japan). CSF concentration of sIL-2R was determined using an ELISA (Cell Free N IL-2R; Kyowa Medex). CSF neopterin level was measured using high-performance liquid chromatography. IFN-γ and six chemokines (CXCL9, CXCL10, CXCL11, CCL3, CCL4, and CCL5) were measured using a CBA (BD Biosciences). The CSF concentrations of three chemokines (CCL17, CCL20, and CCL22) and IL-17A were measured using commercially available ELISA kits (CCL17, CCL20, and CCL22: TECHNE/R&D Systems, Minneapolis, MN USA; IL-17A: Gen-Probe, San Diego, CA USA). All assays were conducted according to the respective manufacturers' instructions.

### Classification system based on the natural history of HAM/TSP

The 53 total HAM/TSP patients without any history of HAM/TSP-targeting treatments were interviewed using a questionnaire (Figure S2) to determine the changes in Osame's Motor Disability Score (OMDS) over time (Figure S3). OMDS is a standardized neurological rating scale as a measure of disability [10] (Figure S1). Based on the changes in OMDS, “deteriorating cases” and “stable cases” were identified in both the Training set and Test set patient cohorts. Patients with deteriorating HAM/TSP were defined as those whose OMDS worsened ≥3 grades over four years and patients with stable HAM/TSP were defined as those whose OMDS remained unchanged or worsened 1 grade over four years. Patients whose OMDS worsened 2 grades over four years were excluded from the patient cohort in order to create a larger gap between the deteriorating and stable patient groups.

### Statistical analysis

GraphPad Prism 5 (GraphPad Software, Inc., La Jolla, CA USA) was used to plot graphs and perform statistical analyses. Differences between the two subject groups were tested using the Mann-Whitney U-test. Receiver operating characteristic (ROC) analysis was performed to examine the sensitivity and specificity of individual biomarkers. For the ROC analyses, an area under the ROC curve (AUC) of 1.0 was used to represent a perfect test with 100% sensitivity and 100% specificity, whereas an area of 0.5 was used to represent random discrimination. Spearman's rank correlation test was employed to investigate the correlation between the four CSF markers (CXCL10, CXCL9, neopterin, and cell count) and the proviral load in PBMCs. To compare the four CSF markers between three groups (HTLV-1-infected control, n = 8; stable HAM/TSP, n = 25; and deteriorating HAM/TSP, n = 20), we used the Kruskal–Wallis test followed by Dunn's post-hoc tests. P-values<0.05 were considered statistically significant.

## Results

### Identification of biomarkers elevated in the blood of HAM/TSP patients

**Figure 1:**
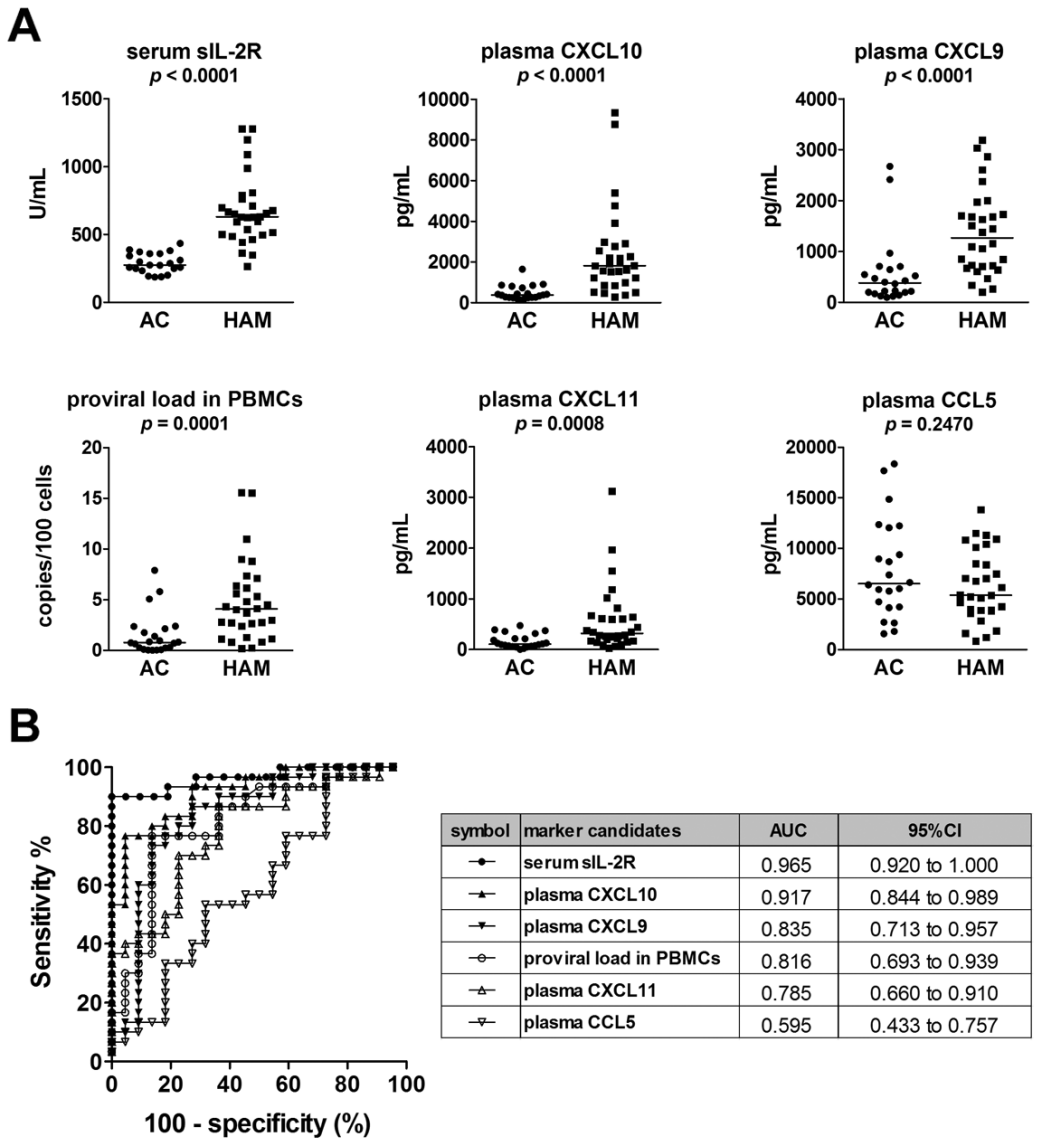
Selection of candidate biomarkers in the blood by comparing HAM/TSP patients and asymptomatic carriers. (**A**) Serum levels of soluble IL-2 receptor (sIL-2R), proviral loads in peripheral blood mononuclear cells (PBMCs), and plasma levels of four chemokines (chemokine (C-X-C motif) ligand (CXCL) 9, CXCL10, CXC11, and chemokine (C-C motif) ligand (CCL) 5) were compared between HAM/TSP patients (HAM; n = 30) and asymptomatic carriers (AC; n = 22). Horizontal bars indicate the median values. The Mann-Whitney *U*-test was used for statistical analysis. (**B**) Receiver operating characteristic (ROC) analysis was employed to assess the sensitivities and specificities of the six markers exhibited in part (A) for discriminating HAM/TSP patients from ACs: greater proximity of the ROC curve to the upper left corner indicates higher sensitivity and specificity of the marker. AUC = area under the ROC curve; 95% CI = 95% confidence interval.

In order to identify candidate blood markers for HAM/TSP, the concentrations of IL-1β, TNF-α, and IFN-γ were measured in plasma samples from four ACs and four HAM/TSP patients. Plasma levels of IL-1β and TNFα were below the detection limits (<2.3 pg/mL and <1.2 pg/mL, respectively) except in one patient with HAM/TSP. Plasma IFN-γ levels showed no significant differences between ACs and HAM/TSP patients (median 10.4 pg/mL and 13.9 pg/mL, respectively). Therefore, these quantities were not measured in additional samples (Figure S1). The proviral DNA load in PBMCs, serum sIL-2R, and plasma levels of the chemokines CXCL9, CXCL10, CXCL11, and CCL5 were also measured in 22 ACs and 30 HAM/TSP patients without any history of immunomodulating treatments, including corticosteroids, IFN-α, and immunosuppressive drugs. The results revealed that serum levels of sIL-2R, plasma levels of CXCL10 and CXCL9, and proviral DNA load in PBMCs were markedly higher in HAM/TSP patients compared to ACs (*p*≤0.0001, Figure 1A). These quantities were then compared using ROC analysis to determine which parameters were superior markers for HAM/TSP. From the results of the ROC analysis, we determined that serum sIL-2R and plasma CXCL10 had the highest potential for distinguishing HAM/TSP patients from ACs with high sensitivity and specificity (area under the ROC curve [AUC]>0.9), followed by plasma CXCL9 and HTLV-1 proviral load in PBMCs (0.8<AUC<0.9) (Figure 1B). Thus, four candidate blood biomarkers were selected for further investigation: serum sIL-2R, plasma CXCL10, plasma CXCL9, and HTLV-1 proviral load in PBMCs.

### Identification of biomarkers elevated in the CSF of HAM/TSP patients

**Figure 2:**
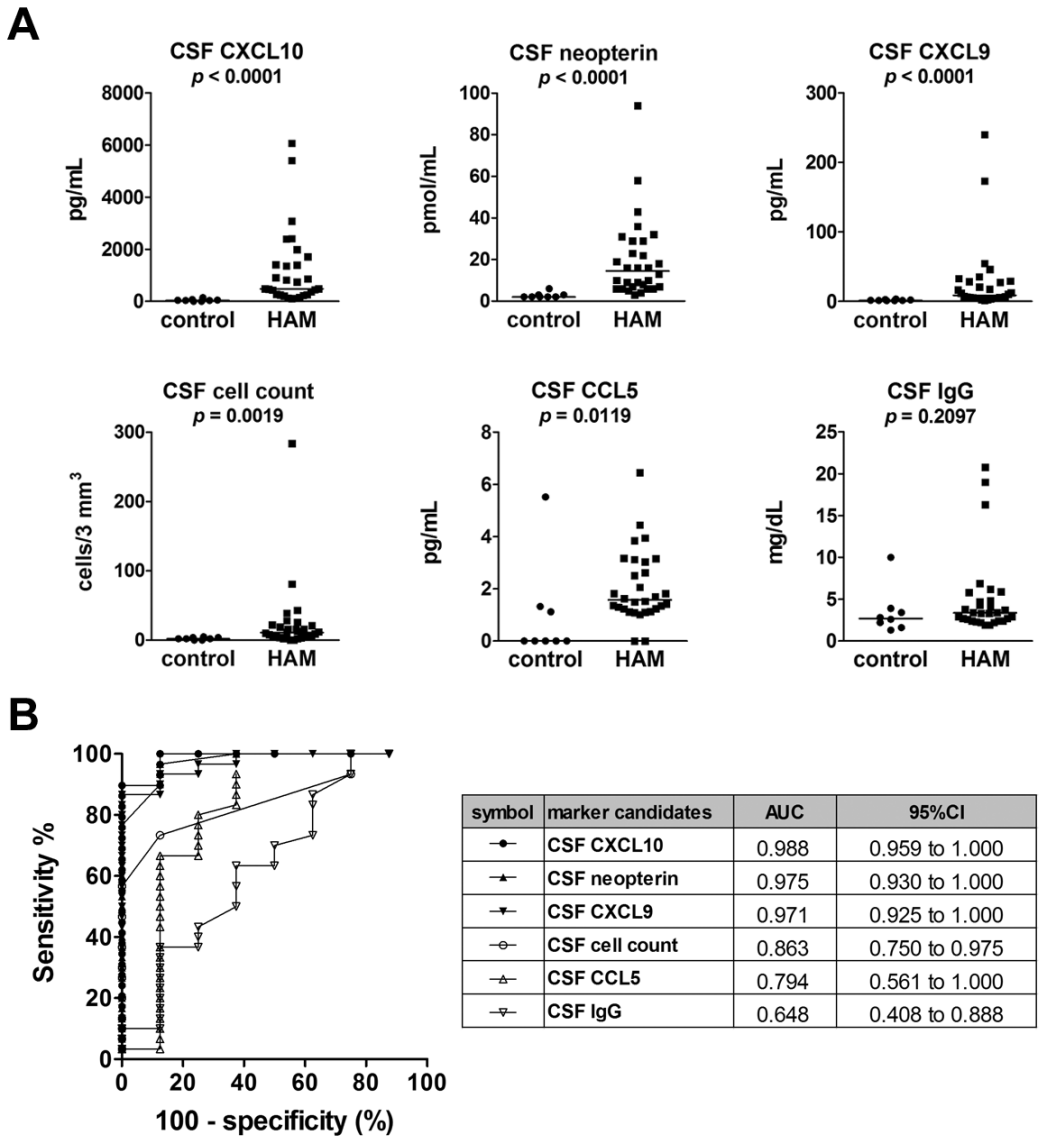
Selection of candidate biomarkers in the cerebrospinal fluid (CSF) by comparing HAM/TSP patients and control subjects. (**A**) CSF levels of total protein, cell count, IgG, neopterin, sIL-2R, and nine chemokines (CCL3, CCL4, CCL5, CXCL9, CXCL10, CXC11, CCL17, CCL20, and CCL22) were measured and compared between HAM/TSP patients (HAM; n = 30) and HTLV-1-infected control subjects (control; n = eight: seven ACs and one ATL patient). Data is shown for the top six CSF markers ranked according to the significance of the difference between the HAM/TSP patients and the control subjects. Horizontal bars indicate the median values. The Mann-Whitney *U*-test was used for statistical analysis. (**B**) ROC analysis was employed to assess the sensitivities and specificities of the six markers exhibited in part (A) for discriminating HAM/TSP patients from controls. AUC = area under the ROC curve; 95% CI = 95% confidence interval.

In order to identify candidate CSF markers for HAM/TSP, elevated levels of various potential markers were screened for in CSF samples from HAM/TSP patients. CSF IL-17A was detectable (>3.0 pg/mL) in only one of eight HAM/TSP patients screened (including six deteriorating-type patients), and the level in this one patient (deteriorating-type) was negligible (4.0 pg/mL). CSF IFN-γ was detectable (>1.8 pg/mL) in only 3 of 10 HAM/TSP patients screened (six deteriorating patients), and the levels in all three were negligible (range 3.3–4.2 pg/mL). Therefore, these cytokines were not measured in additional patients. Total protein, cell count, IgG, neopterin, sIL-2R, and nine chemokines (CXCR3 ligands: CXCL9, CXCL10, and CXCL11; CCR5 ligands: CCL3, CCL4, and CCL5; CCR4 ligands: CCL17 and CCL22; CCR6 ligand: CCL20) were also measured in the CSF of 30 untreated HAM/TSP patients and in eight HTLV-1-infected control subjects (seven ACs and one patient with smoldering ATL). The results indicated that CSF levels of CXCL10, neopterin, and CXCL9 were remarkably higher in HAM/TSP patients compared to control subjects (*p*<0.0001 overall, Figures 2A and S4) and that CSF levels of cell count and CCL5 were less so but still significantly higher (*p* = 0.0019 and *p* = 0.0119, respectively; Figure 2A). By contrast, there were no differences in the CSF levels of IgG and total protein between HAM/TSP patients and control subjects, and CSF sIL-2R levels were only detectable in a single HAM/TSP patient (data not shown). ROC analysis showed that the CSF levels of CXCL10, neopterin, CXCL9, and CSF cell count could be used to relatively accurately distinguish HAM/TSP patients from control subjects (AUC>0.8) (Figure 2B). Therefore, these four CSF markers were selected as candidates for further investigation. It should be noted that the sensitivity of CSF cell count was very low (36.7%) when compared to the other three: CXCL10 (83.3%), CXCL9 (86.7%), and neopterin (76.7%) (Figure S5).

### Identification of biomarkers correlated with rate of HAM/TSP disease progression

**Figure 3:**
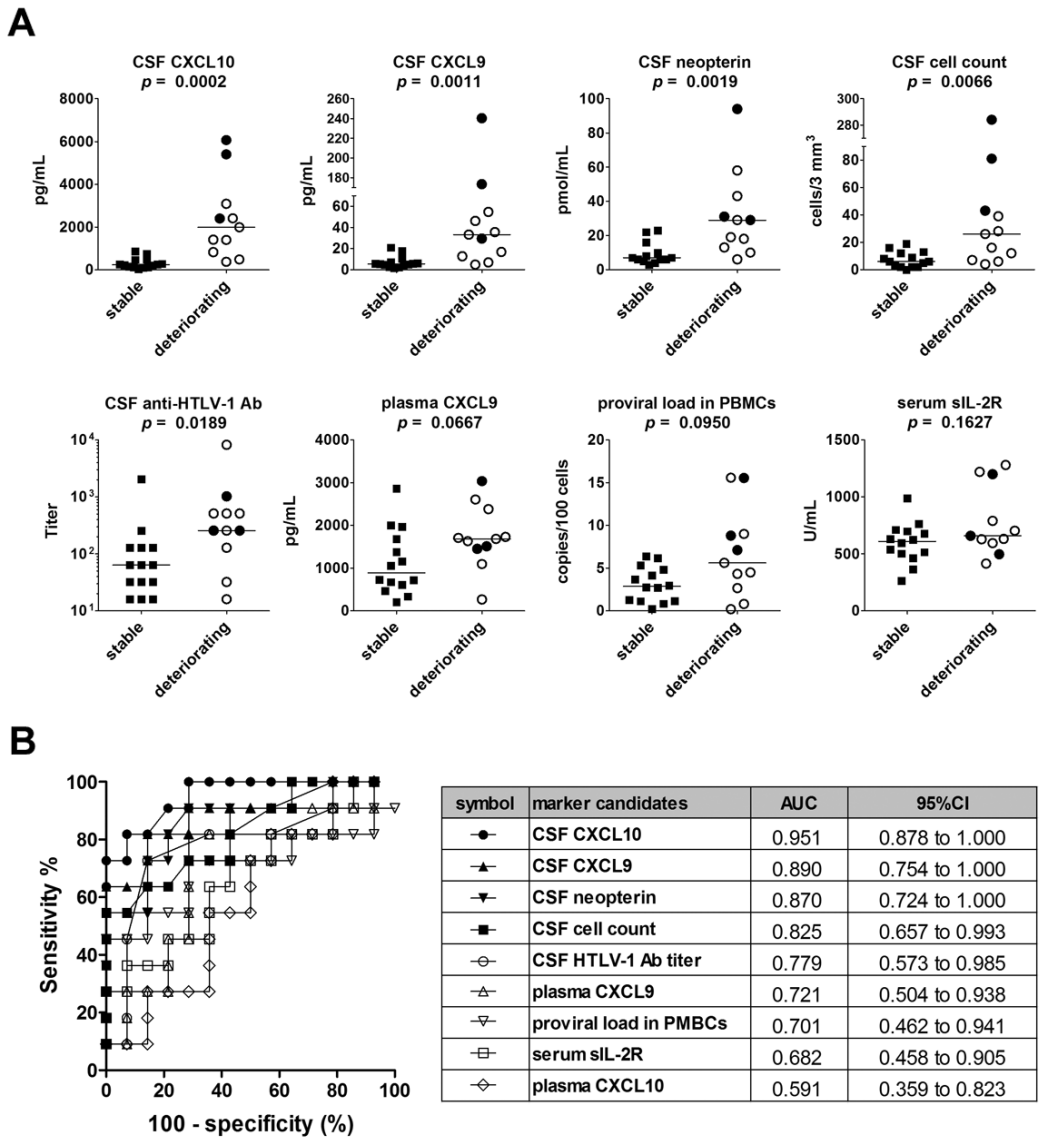
Identification of biomarkers associated with clinical progression of HAM/TSP. (**A**) Five CSF marker candidates (CXCL10, CXCL9, neopterin, cell count, and anti-HTLV-1 antibody titer) and four blood marker candidates (proviral load in PBMCs, serum sIL-2R, plasma CXCL9, and plasma CXCL10) were compared among a cohort of patients called the Training Set (deteriorating HAM/TSP, n = 11; stable HAM/TSP, n = 14). Data is shown for the top eight CSF markers ranked according to the significance of the difference between the deteriorating and stable subjects. Black circles indicate patients with particularly rapidly progressive HAM/TSP. Horizontal bars indicate the median values. The Mann-Whitney *U*-test was used for statistical analysis. (**B**) ROC analysis was employed to assess the sensitivities and specificities of the nine markers listed above for discriminating deteriorating HAM/TSP patients from stable patients. AUC = area under the ROC curve; 95% CI = 95% confidence interval.

In short, we selected nine markers: eight markers chosen based on the analyses described above and CSF anti-HTLV-1 antibody titer, which is a known diagnostic marker for HAM/TSP. To determine which biomarkers were associated with HAM/TSP disease progression, the levels of these nine markers were compared between the deteriorating and stable HAM/TSP patient groups (see Methods for definitions of deteriorating and stable). The results revealed that all five CSF markers were significantly higher in the deteriorating group compared to the stable group (Figure 3A), but that none of the four blood markers, including proviral load, were significantly different between the two groups. The deteriorating group included three patients with particularly rapidly progressive HAM/TSP, defined as those who had been confined to wheelchairs (OMDS: ≥ grade 6) within two years after the onset of symptoms [13], [14] (black circles in Figures 3A and S3B). These rapid progressors exhibited high levels of the CSF markers and high proviral loads. ROC analysis revealed that the levels of the CSF markers (CXCL10, CXCL9, neopterin, and cell count), but not anti-HTLV-1 antibody titer, distinguished clearly between patients with deteriorating HAM/TSP and stable HAM/TSP (AUC>0.8, Figure 3B).

### Validation of nine candidate biomarkers using the Test Set

**Figure 4:**
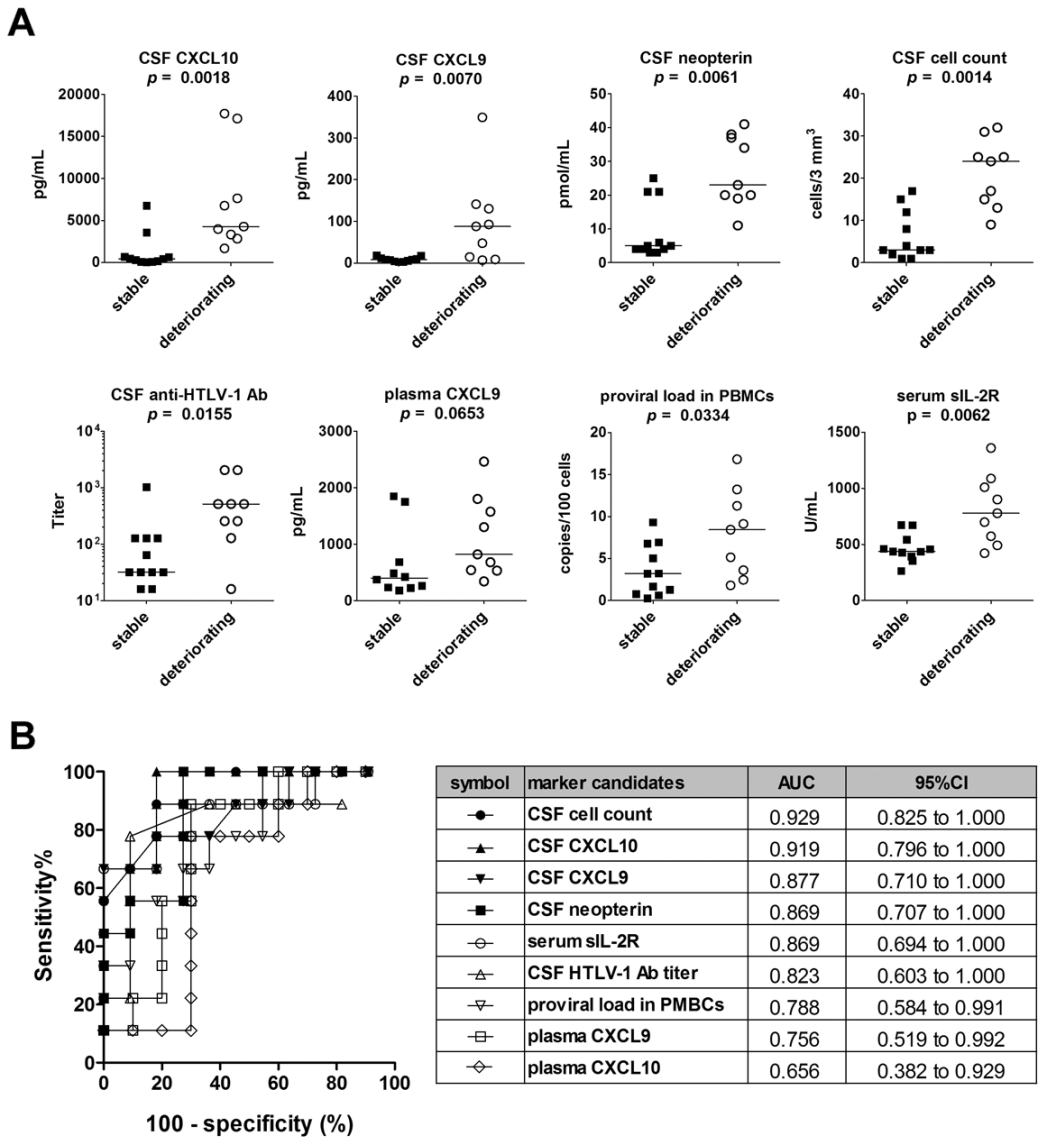
Validation of potential markers using the Test Set. (**A**) Five CSF marker candidates (CXCL10, CXCL9, neopterin, cell count, and anti-HTLV-1 antibody titer) and four blood marker candidates (proviral load in PBMCs, serum sIL-2R, plasma CXCL9, and plasma CXCL10) were compared among a second cohort of patients called the Test Set (deteriorating HAM/TSP, n = 9; stable HAM/TSP, n = 11). Data is shown for the top eight CSF markers ranked according to the significance of the difference between the deteriorating and stable subjects. Horizontal bars indicate the median values. The Mann-Whitney *U*-test was used for statistical analysis. (**B**) ROC analysis was employed to assess the sensitivities and specificities of the nine markers listed above for discriminating deteriorating HAM/TSP patients from stable patients. AUC = area under the ROC curve; 95% CI = 95% confidence interval.

To validate the results obtained using the Training Set, the same nine markers were compared between deteriorating and stable patients using the Test Set (a second cohort of 23 HAM/TSP patients that had not undergone HAM/TSP-targeting treatment). As shown in Figure 4A, the results indicated that the levels of five CSF markers, proviral load in PBMCs, and serum sIL-2R were significantly higher in deteriorating cases than in stable cases. Among them, CSF levels of CXCL10, CXCL9, neopterin, and CSF cell count exhibited particularly high sensitivities and specificities for detecting the deteriorating HAM/TSP cases in the Test set as well as Training set (AUC>0.8, Figures 4B and S1).

### Demographic and clinical characteristics of the subjects

The demographics of the HAM/TSP patients versus the control subjects for both the blood tests and CSF analyses were compared and evaluated for statistical significance (Table S1). There were no significant differences in age or gender distribution between the HAM/TSP patients and either control subject group.

Similarly, the demographic and clinical characteristics of stable versus deteriorating HAM/TSP subjects in both the Training and Test sets are shown in Tables 2 and 3, respectively. There were no significant differences in age or gender distribution among either set, but deteriorating patients in both sets were significantly older at disease onset and had been living with the disease for shorter periods of time. Deteriorating patients in the Training set scored higher OMDS values than their stable counterparts (p<0.01), but there was no such significant difference in the Test set.

**Figure 5:**
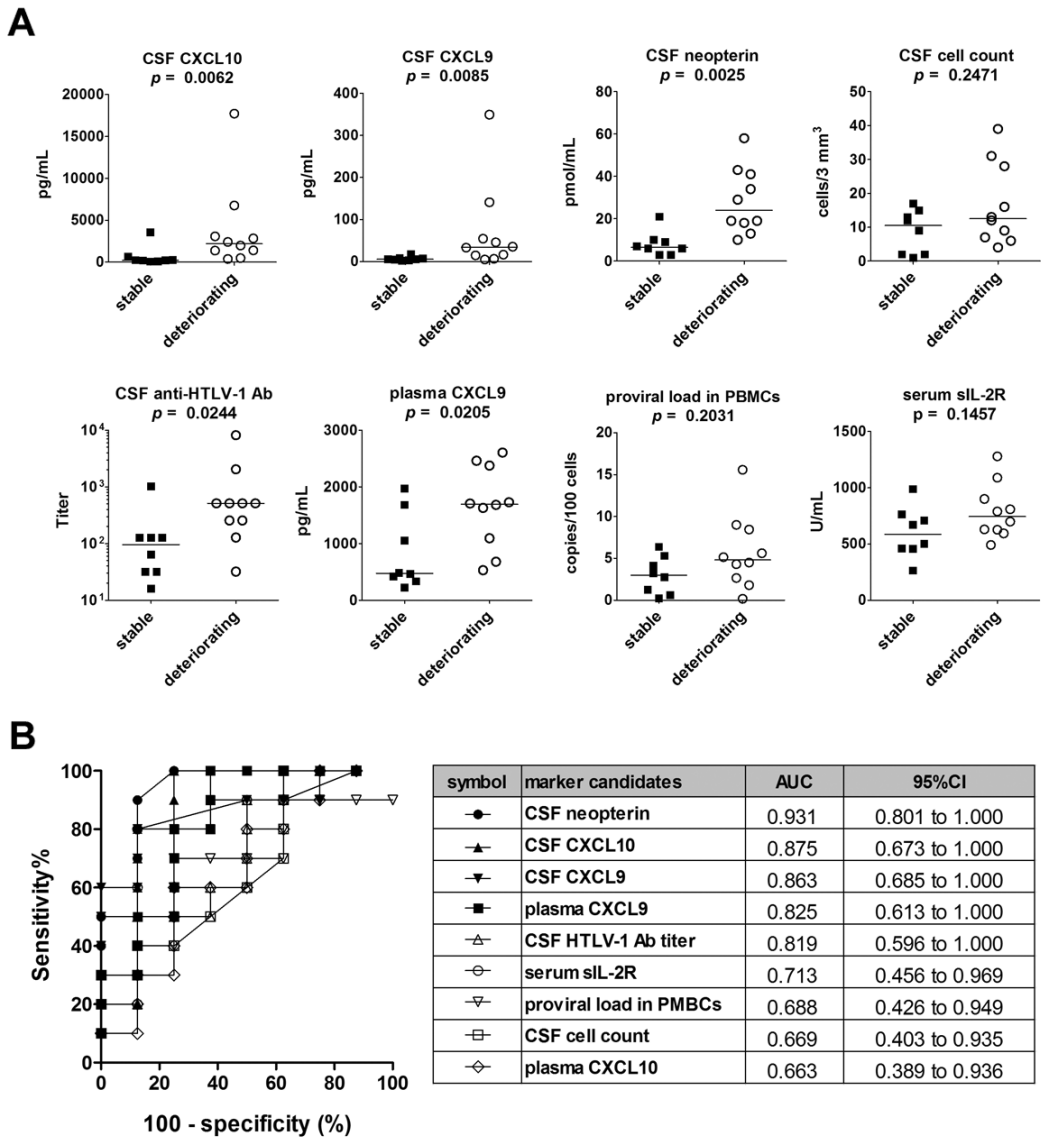
Comparison of potential markers in stable and deteriorating HAM/TSP patients with similar disease durations. (**A**) Five CSF marker candidates (CXCL10, CXCL9, neopterin, cell count, and anti-HTLV-1 antibody titer) and four blood marker candidates (proviral load in PBMCs, serum sIL-2R, plasma CXCL9, and plasma CXCL10) were compared among all patients from both the Training and Test Sets pooled together with similar disease durations (range: 7–13 years; no significant difference in duration between stable (n = 8) and deteriorating (n = 10) groups). Data is shown for the top eight CSF markers ranked according to the significance of the difference between the deteriorating and stable subjects. Horizontal bars indicate the median values. The Mann-Whitney *U*-test was used for statistical analysis. (**B**) ROC analysis was employed to assess the sensitivities and specificities of the nine markers listed above for discriminating deteriorating HAM/TSP patients from stable patients while controlling for disease duration. AUC = area under the ROC curve; 95% CI = 95% confidence interval.

To investigate the potential influence of disease duration as a secondary variable, a new test group was created containing only those patients for whom the disease onset date was 7–13 years prior to the sample collection day. Patients fitting this criterion were selected from the 53 total available from both the Training and Test sets: eight stable patients and ten deteriorating patients; we confirmed that there was no significant difference in disease duration between these two groups. The results remained consistent with our previous findings: CSF CXCL10, CXCL9, and neopterin were all elevated in deteriorating patients with respect to stable patients (p<0.01, Figure 5).

### Follow-up mini-study on biomarker levels over time

Four stable HAM/TSP patients were left completely untreated and followed for a period of three to five years. Within this time, one patient rose one grade on the OMDS scale, and the other three experienced no change in OMDS grade at all. The levels of CSF CXCL10 and neopterin remained consistently low over time (Figure S6).

## Discussion

To date, there have been few well-designed studies that have evaluated the relationship between biomarkers and HAM/TSP disease progression. In a previous retrospective study with 100 untreated HAM/TSP patients, a significant association was demonstrated to exist between higher HTLV-1 proviral load in PBMCs and poor long-term prognosis; however, the predictive value of high proviral load appeared to be too low to qualify it as a marker for disease progression in clinical practice [32]. Here we conducted a retrospective study to compare for the first time the relationships of PBMC proviral load and several inflammatory biomarker candidates to disease progression in untreated HAM/TSP patients.

In this study, elevated CSF cell count, neopterin concentration, and CSF levels of CXCL9 and CXCL10 were well-correlated with disease progression over the four year period under study, better even than HTLV-1 proviral load in PBMCs (Figures 3 and 4). As CSF pleocytosis, CSF CXCL10, CSF CXCL9, and CSF neopterin are known indicators of inflammation in the central nervous system [33], [34], our findings indicate that the rate of HAM/TSP progression is more closely reflected by the amount of inflammatory activity in the spinal cord than by the PBMC proviral load. However, we also found a significant correlation between PBMC proviral load and the levels of the CSF markers identified in this study (Figure S7), indicating that a higher PBMC proviral load does indeed suggest more inflammation in the spinal cord and therefore a poorer long-term prognosis. These findings are consistent with the theory that HAM/TSP is the result of an excess of inflammatory mediators caused by the presence of HTLV-1-infected T-cells [35]–[37].

The HTLV-1 proviral load in the CSF as well as the ratio of the proviral load in the CSF to that in PBMCs have been reported to be effective for discriminating HAM/TSP patients from ACs or multiple sclerosis patients infected with HTLV-1 [38], [39]. Some researchers have suggested that these values might be associated with the rate of disease progression, but there has been only one small cohort study and one case report investigating this point, and so the significance of this experimental evidence is still questionable [40], [41]. In addition to statistical validation with multiple, larger cohorts, it would also be beneficial to use precise definitions for progressive versus stable patients, as we have done in this study. Although the volume of CSF available per sample was too limited to measure CSF proviral load in the present study, we plan to incorporate CSF proviral load in a future prospective study and compare its usefulness to that of other biomarker candidates.

From our results, we concluded that of the potential biomarkers under study, CXCL10, CXCL9, and neopterin are the most fit for determining the level of spinal cord inflammation, and thus the most fit for predicting disease progression in HAM/TSP patients. Although the CSF cell count is an easily measurable inflammatory marker, it is not sensitive enough to reliably detect the level of spinal cord inflammation. Numerous patients with CSF cell counts within the normal range exhibited high levels of other inflammatory markers, such as neopterin and CXCL10 (Figure S5). In fact, it has been reported that CSF pleocytosis is present in only approximately 30% of HAM/TSP patients [42]. Furthermore, in our study, there was no significant difference in CSF cell count between the control subjects and the stable HAM/TSP patients (Figure S8).

We also explored the possibility of combining multiple biomarkers via multiple logistic regression to form a combination more sensitive and specific than individual markers, but the results indicated that there is not much to be gained from combinations (data not shown).

While there were no significant demographic differences between subject groups, the clinical characteristics of stable versus deteriorating HAM/TSP patients of course differed widely (Tables 2, 3, and S2). We confirmed the already well-reported statistic that deteriorating patients experience HAM/TSP onset relatively late in life [12], [14], [20] ; our data also reflected the short disease duration expected of deteriorating patients, who by definition progress through the disease more rapidly than their stable counterparts. As patients in all groups were of similar age at sample collection, the significant difference in age of onset should not have any impact on our findings. However, it was necessary to consider the possibility that those patients in a later stage of the disease (i.e. those listed with longer disease durations) might possess elevated or diminished biomarker levels regardless of rate of disease progression. We confirmed that this difference in disease duration was not a confounding factor in our selection of candidate biomarkers by comparing stable and deteriorating HAM/TSP patients with similar disease durations (7–13 years), and we were able to obtain results consistent with our earlier findings (Figure 5). Finally, the OMDS values for the stable and deteriorating patient groups in the Test set were perfectly identical, eliminating the need to consider the possibility that the biomarkers could have been elevated according to disease severity regardless of rate of progression.

The main limitation of our retrospective study is that our samples were collected from patients at the end of the four year period during which the extent of progression was analyzed as opposed to the beginning of the four year period, which would have been optimal for directly measuring their prognostic powers. Of course, the patients with severe HAM/TSP symptoms began undergoing treatment soon after sample collection, rendering any observations on disease course after sample collection un-useable for analysis in this study. While this situation is non-ideal, we hypothesize that biomarker levels in a given patient do not substantially change over a few years' time. We were actually able to monitor the biomarker levels of four untreated HAM/TSP patients over 3–5 years, and the levels remained relatively stable in all four subjects over time (Figure S6), supporting our hypothesis. However, these were all stable HAM/TSP patients (hence the lack of treatment), and so we cannot rule out the possibility that biomarker levels in untreated deteriorating patients may dramatically rise, fall, or fluctuate. The results of the analysis of patients with similar disease durations (Figure 5) also support our hypothesis that disease duration is not an important determinant of biomarker levels, but it is of course not conclusive. We expect that a prospective study in the future will reveal the answer to this question.

The results of this study indicate that CXCL9 and/or CXCL10 may play a key role in the pathogenesis of HAM/TSP by recruiting more inflammatory cells to the spinal cord lesions. In this study, we measured the levels of the chemokines in the CSF that might play a part in inducing the migration of T-helper (Th) cells. CD4^+^ Th cells differentiate from naïve T-cells to members of the Th subset (e.g., Th1, Th2, Th17, or Treg cells), and each one expresses its own characteristic chemokine receptors [43]. Usually, Th1 cell express CCR5/CXCR3 receptors, Th2 and Treg cells express CCR4, and Th17 express CCR6. Interestingly, CCR4 ligands (CCL17 and, CCL22) and the CCR6 ligand (CCL20) were not detected in the CSF of HAM/TSP patients. Moreover, of the CCR5 ligands, only CCL5 was elevated, but only slightly, and there was no association with rate of disease progression. Of the CXCR3 ligands, only CXCL9 and CXCL10 were correlated with the rate of disease progression. These results show that the pathology of HAM/TSP is unique among immune disorders in that, unlike other inflammatory disorders such as multiple sclerosis or rheumatoid arthritis that exhibit Th17 as well as Th1 involvement, the chemokine involvement in HAM/TSP is Th1-dominant. In a previous study, cytokines produced by HTLV-1-infected T-cells in HAM/TSP patients were analyzed, and the results showed that IFN-γ was elevated and IL-17 reduced [43], [44]. Taken together, the results of these studies indicate that the characteristics of HTLV-1-infected T-cells themselves may be responsible for the Th1-dominant chemokine production observed in HAM/TSP. Also, these results suggest that the CXCR3-ligand (CXCL9 and CXCL10) interactions play an important role in the pathophysiology of HAM/TSP. Recently it was established that these CXCR3-ligand interactions are extremely important for the pathogenesis of several neurological disorders [33]. Therefore, future research on the significance of these interactions in the pathogenic process of HAM/TSP will be important for clarifying the suitability of CXCL9 and CXCL10 as biomarkers or therapeutic targets.

In conclusion, in this retrospective study, we have demonstrated that CSF levels of CXCL10, CXCL9, and neopterin are promising candidate prognostic biomarkers for HAM/TSP. These biomarkers may provide a means for the early identification of patients at increased risk of debilitating disease progression, those that may need anti-inflammatory therapies to limit or prevent this, and for evaluating the efficacy of such therapies. This initial identification of prognostic biomarkers for HAM/TSP should be followed by a future multicenter prospective clinical study.

## Supporting Information

Figure S1 **Diagram illustrating the biomarker selection process.** A total of 26 biomarker candidates including 9 in the blood and 17 in the CSF underwent the following selection processes: 1) pre-screening of the cytokines for presence in HAM/TSP patients, 2) selection for markers elevated in HAM/TSP patients with respect to controls (AUC>0.8), 3) selection for markers elevated in deteriorating HAM/TSP patients with respect to stable patients (AUC>0.8) in a cohort termed the Training Set, 4) validation of the selected markers by evaluating again (AUC>0.8) in a second cohort termed the Test Set. The darkening of an arrow's color represents that marker's failure to meet the selection criteria, and the termination of an arrow indicates that no further testing was conducted for that marker. CYT = cytokine, HTLV-1 PVL = HTLV-1 proviral load, Ab Titer = anti-HTLV-1 antibody titer, AUC = area under the ROC curve.
(TIF)

Figure S2 **Questionnaire on the development of motor disability over time as measured using Osame's Motor Disability Score (OMDS).** The first and second columns indicate the OMDS numerical value and description, respectively. Doctors interviewed the patients and filled in the table according to the following instructions: in the bottom row, write the ages at which symptoms listed to the left first appeared, and above the age check the box in the row corresponding to the symptom.
(TIF)

Figure S3 **Rate of disease progression in HAM/TSP patients without any history of HAM/TSP-targeting treatment.** Each line illustrates the change in OMDS over time for an individual patient after disease onset for (**A**) all patients in the Training Set (n = 30) and (**B, left**) only deteriorating patients (n = 11) including three particularly rapidly progressive patients (shown as solid black circles) and (**B, right**) only stable patients (n = 14).
(TIF)

Figure S4 **Comparison of CSF levels of nine chemokines in control subjects and HAM/TSP patients.** The CSF levels of nine chemokines (CCR5 ligands: CCL3, CCL4, and CCL5; CXCR3 ligands: CXCL9, CXCL10, and CXCL11; CCR4 ligands: CCL17 and CCL22; CCR6 ligand: CCL20) were compared between control subjects (control; n = 8) and HAM/TSP patients (HAM; n = 30). Horizontal bars indicate median values. The Mann-Whitney *U*-test was used for statistical analysis.
(TIF)

Figure S5 **Low sensitivity of CSF cell count for detection of HAM/TSP.** (**A**) Sensitivities of four potential CSF markers for detection of HAM/TSP. For CSF CXCL10, CXCL9, and neopterin, dotted lines indicate reference values, defined as mean for control subjects +3 standard deviations. For CSF cell count, the dotted line represents the pre-established reference value of 15/3 mm^3^. The sensitivity of CSF cell count was much lower than those of the other CSF markers. (**B**) Direct comparison of the sensitivities of CSF cell count and the other three CSF markers. The horizontal dotted lines all represent the reference value for CSF cell count (≤15/3 mm^3^), and each vertical dotted line indicates the reference value for each of the other CSF markers. With these lines drawn, one can see in the shaded area the numerous patients with CSF cell counts within the normal range but abnormally high levels of each of the other inflammatory markers, thus directly illustrating the comparatively low sensitivity of CSF cell count.
(TIF)

Figure S6 **Changes in levels of CSF markers and OMDS over time in four untreated HAM/TSP patients.** The three graphs illustrate the changes over time in CSF CXCL10 (top), neopterin (middle), and OMDS (bottom) for four untreated stable HAM/TSP patients. The patients were observed for 60 months (No. 1), 56 months (No. 2), 49 months (No. 3), and 39 months (No. 4).
(TIF)

Figure S7 **Significant positive correlation between the proviral load in PBMCs and four CSF markers.** HTLV-1 proviral load in PBMCs was compared with the levels of each of four CSF markers (CXCL10, CXCL9, neopterin, and cell count) in HAM/TSP patients (n = 53). Data analysis was performed using the Spearman's rank correlation test.
(TIF)

Figure S8 **Significant higher CSF levels of CXCL10, CXCL9, and neopterin even in stable HAM/TSP compared to controls.** The levels of four CSF markers (CXCL10, CXCL9, neopterin, and cell count) were compared among three groups (HTLV-1-infected controls, n = 8; stable HAM/TSP patients, n = 25; and deteriorating HAM/TSP patients, n = 20) assembling patients from both Training and Test Sets combined. The horizontal bar indicates the median value for each group. Statistical analysis was performed using the Kruskal–Wallis test followed by Dunn's post-hoc tests. ns: not significant, * *P*<0.05, *** *P*<0.001.
(TIF)

Table S1 **Demographics of HAM/TSP patients and control subjects.** There were no significant differences in the demographics of HAM/TSP patients versus control subjects.
(DOCX)

Table S2 **Demographics and clinical characteristics of HAM/TSP patients (Training set + Test Set).** Among the HAM/TSP patients from the Training and Test Sets pooled together, deteriorating patients experienced disease onset significantly later in life and had lived with the disease for shorter periods.
(DOCX)
