# Supplementary figures and images for: CSF CXCL10, CXCL9, and Neopterin as Candidate Prognostic Biomarkers for HTLV-1-Associated Myelopathy/Tropical Spastic Paraparesis
Source: PLoS Negl Trop Dis. 2013 Oct 10;7(10):e2479. doi: 10.1371/journal.pntd.0002479 (PMC3794911; doi:10.1371/journal.pntd.0002479)

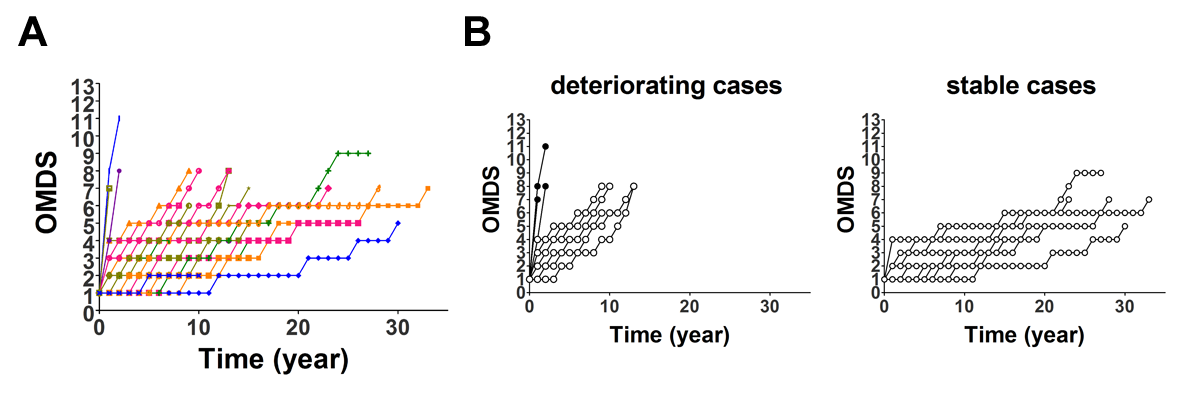

Supplement: Figure S3 — Rate of disease progression in HAM/TSP patients without any history of HAM/TSP-targeting treatment. Each line illustrates the change in OMDS over time for an individual patient after disease onset for (A) all patients in the Training Set (n = 30) and (B, left) only deteriorating patients (n = 11) including three particularly rapidly progressive patients (shown as solid black circles) and (B, right) only stable patients (n = 14). (TIF) [file pntd.0002479.s003.tif]

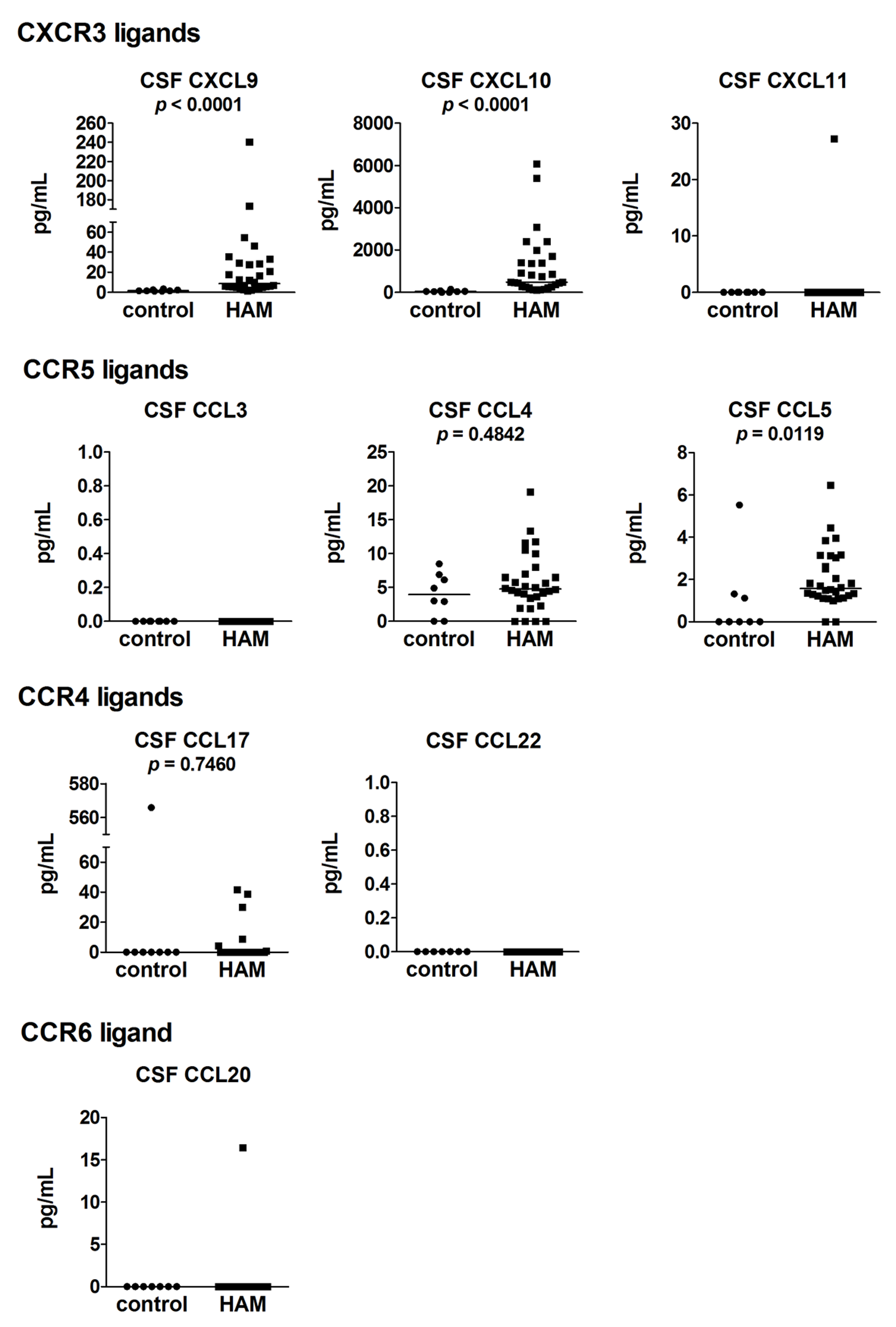

Supplement: Figure S4 — Comparison of CSF levels of nine chemokines in control subjects and HAM/TSP patients. The CSF levels of nine chemokines (CCR5 ligands: CCL3, CCL4, and CCL5; CXCR3 ligands: CXCL9, CXCL10, and CXCL11; CCR4 ligands: CCL17 and CCL22; CCR6 ligand: CCL20) were compared between control subjects (control; n = 8) and HAM/TSP patients (HAM; n = 30). Horizontal bars indicate median values. The Mann-Whitney U-test was used for statistical analysis. (TIF) [file pntd.0002479.s004.tif]

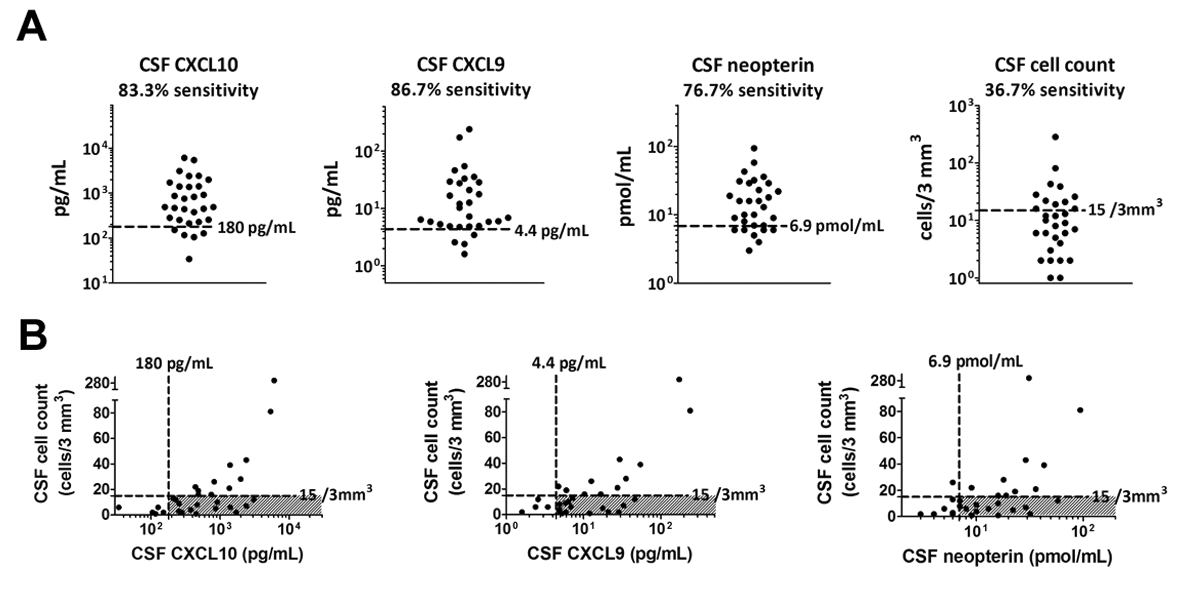

Supplement: Figure S5 — Low sensitivity of CSF cell count for detection of HAM/TSP. (A) Sensitivities of four potential CSF markers for detection of HAM/TSP. For CSF CXCL10, CXCL9, and neopterin, dotted lines indicate reference values, defined as mean for control subjects +3 standard deviations. For CSF cell count, the dotted line represents the pre-established reference value of 15/3 mm3. The sensitivity of CSF cell count was much lower than those of the other CSF markers. (B) Direct comparison of the sensitivities of CSF cell count and the other three CSF markers. The horizontal dotted lines all represent the reference value for CSF cell count (≤15/3 mm3), and each vertical dotted line indicates the reference value for each of the other CSF markers. With these lines drawn, one can see in the shaded area the numerous patients with CSF cell counts within the normal range but abnormally high levels of each of the other inflammatory markers, thus directly illustrating the comparatively low sensitivity of CSF cell count. (TIF) [file pntd.0002479.s005.tif]

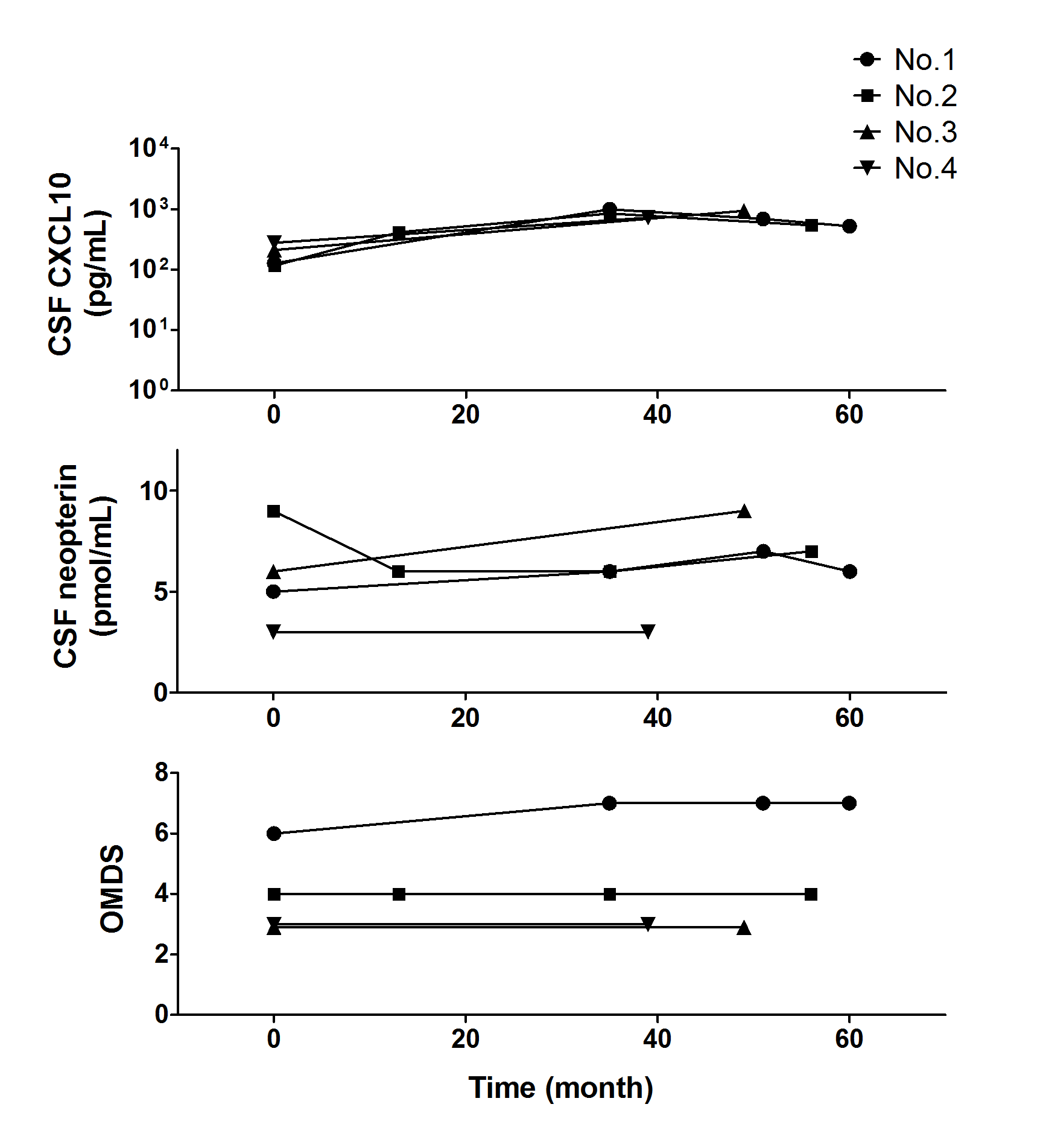

Supplement: Figure S6 — Changes in levels of CSF markers and OMDS over time in four untreated HAM/TSP patients. The three graphs illustrate the changes over time in CSF CXCL10 (top), neopterin (middle), and OMDS (bottom) for four untreated stable HAM/TSP patients. The patients were observed for 60 months (No. 1), 56 months (No. 2), 49 months (No. 3), and 39 months (No. 4). (TIF) [file pntd.0002479.s006.tif]

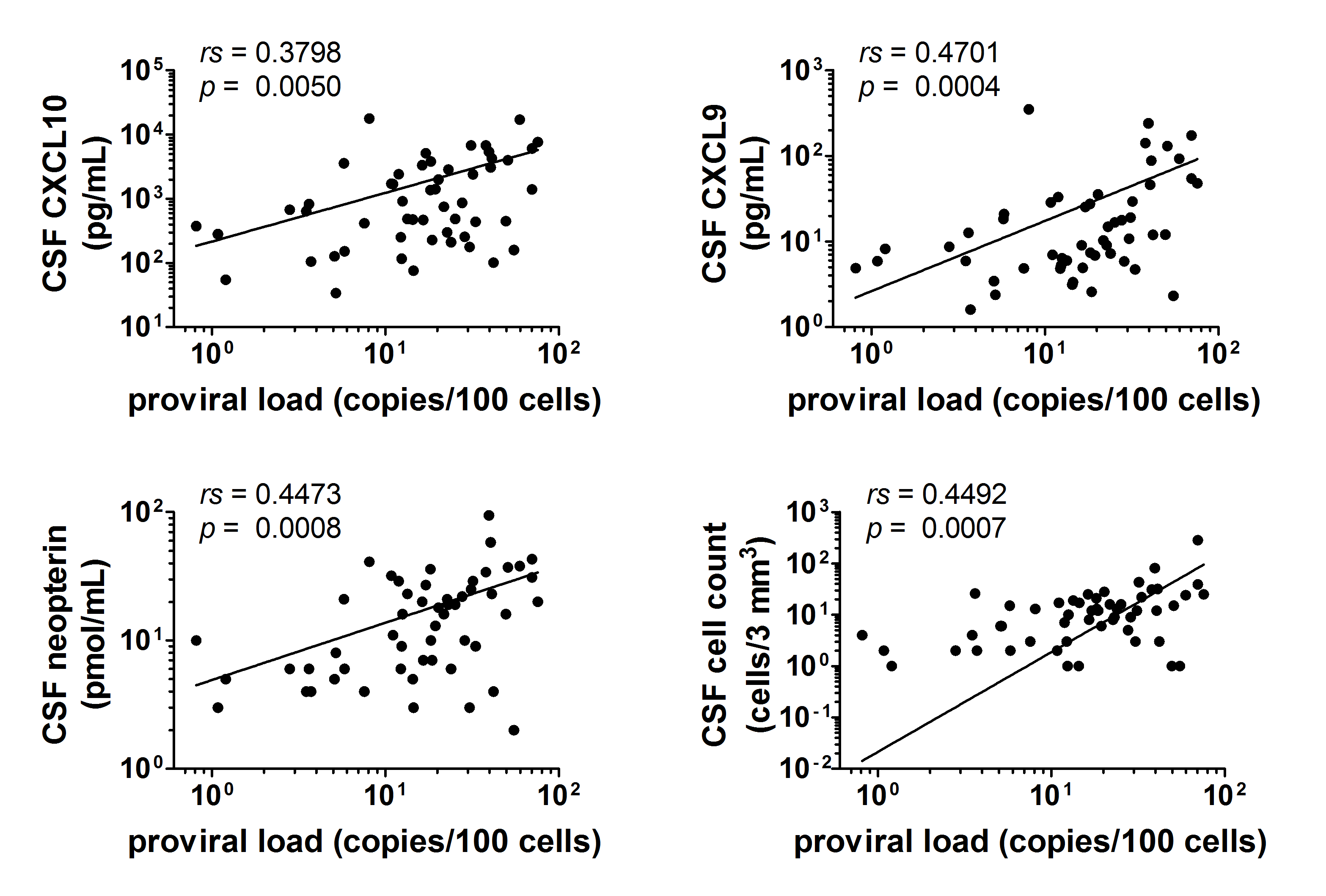

Supplement: Figure S7 — Significant positive correlation between the proviral load in PBMCs and four CSF markers. HTLV-1 proviral load in PBMCs was compared with the levels of each of four CSF markers (CXCL10, CXCL9, neopterin, and cell count) in HAM/TSP patients (n = 53). Data analysis was performed using the Spearman's rank correlation test. (TIF) [file pntd.0002479.s007.tif]

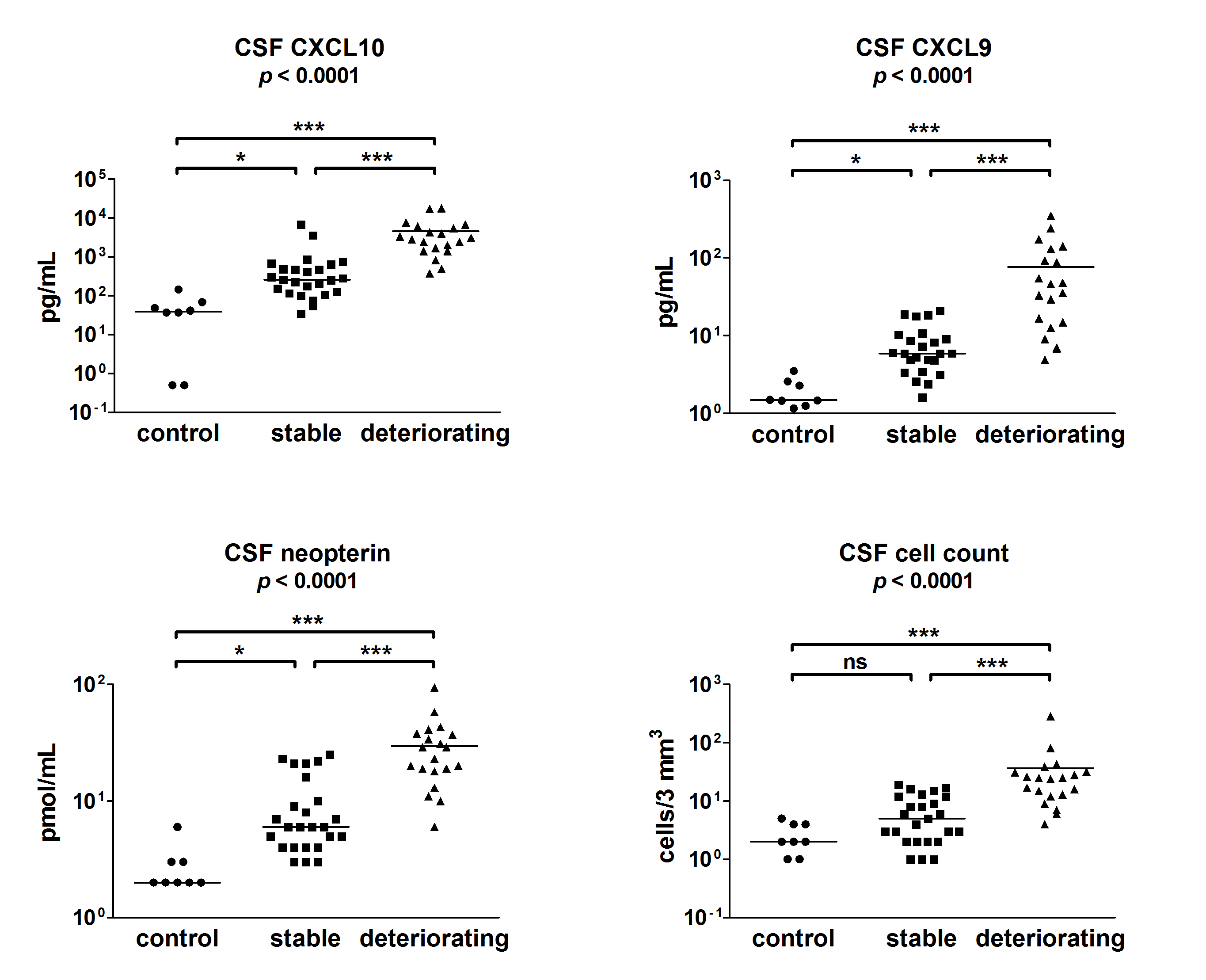

Supplement: Figure S8 — Significant higher CSF levels of CXCL10, CXCL9, and neopterin even in stable HAM/TSP compared to controls. The levels of four CSF markers (CXCL10, CXCL9, neopterin, and cell count) were compared among three groups (HTLV-1-infected controls, n = 8; stable HAM/TSP patients, n = 25; and deteriorating HAM/TSP patients, n = 20) assembling patients from both Training and Test Sets combined. The horizontal bar indicates the median value for each group. Statistical analysis was performed using the Kruskal–Wallis test followed by Dunn's post-hoc tests. ns: not significant, * P<0.05, *** P<0.001. (TIF) [file pntd.0002479.s008.tif]
