# Supplementary material for: CSF CXCL10, CXCL9, and Neopterin as Candidate Prognostic Biomarkers for HTLV-1-Associated Myelopathy/Tropical Spastic Paraparesis
Source: PLoS Negl Trop Dis. 2013 Oct 10;7(10):e2479. doi: 10.1371/journal.pntd.0002479 (PMC3794911; doi:10.1371/journal.pntd.0002479)
Supplement: Table S1 — Demographics of HAM/TSP patients and control subjects. There were no significant differences in the demographics of HAM/TSP patients versus control subjects. (DOCX) [file pntd.0002479.s009.docx]

**Table S1.** Demographics of HAM/TSP patients and control subjects

|  | For blood markers | |  | For CSF markers | |  |
| --- | --- | --- | --- | --- | --- | --- |
|  | AC | HAM/TSP |  | HTLV-1-infected control subjects | HAM/TSP |  |
|  | n =22 | n =30 | *p*-value | n =8 | n =30 | *p*-value |
| **Demographics** | | | | | |  |
| Age, y^*^ | 54 [39–85] | 58 [37–75] | 0.0813^†^ | 56 [31–69] | 58 [37–75] | 0.2368^†^ |
| Female sex | 68.2% | 80.0% | 0.3533^‡^ | 62.5% | 80.0% | 0.3631^‡^ |

There were no significant differences in the demographics of HAM/TSP patients versus control subjects.

*Data are expressed as median [range]　　† By Mann-Whitney test ‡By Fisher’s exact test

AC = asymptomatic carriers
