# Supplementary material for: CSF CXCL10, CXCL9, and Neopterin as Candidate Prognostic Biomarkers for HTLV-1-Associated Myelopathy/Tropical Spastic Paraparesis
Source: PLoS Negl Trop Dis. 2013 Oct 10;7(10):e2479. doi: 10.1371/journal.pntd.0002479 (PMC3794911; doi:10.1371/journal.pntd.0002479)
Supplement: Table S2 — Demographics and clinical characteristics of HAM/TSP patients (Training set + Test Set). Among the HAM/TSP patients from the Training and Test Sets pooled together, deteriorating patients experienced disease onset significantly later in life and had lived with the disease for shorter periods. (DOCX) [file pntd.0002479.s010.docx]

**Table S2.** Demographics and clinical characteristics of HAM/TSP patients (Training set + Test Set)

|  | Total | Stable HAM/TSP | Deteriorating HAM/TSP |  |
| --- | --- | --- | --- | --- |
|  | n = 53 | n =25 | n = 20 | *p*-value^*^ |
| **Demographics** |  |  |  |  |
| Age, y^**^ | 58 [22–75] | 55 [22–75] | 61 [48–72] | 0.1280^†^ |
| Female sex | 79.2% | 72.0% | 85.0% | 0.4728^‡^ |
|  |  |  |  |  |
| **Clinical characteristics** | |  |  |  |
| Age of onset, y^**^ | 45 [12–70] | 34 [14–70] | 57 [39–70] | < 0.0001^†^ |
| Disease duration, y^**^ | 10 [1–41] | 19 [5–41] | 8 [1–14] | < 0.0001^†^ |
| OMDS^**^ | 5 [2–11] | 5 [2–9] | 6 [4–11] | 0.0749^†^ |

Among the HAM/TSP patients from the Training and Test Sets pooled together, deteriorating patients experienced disease onset significantly later in life and had lived with the disease for shorter periods.

*Stable HAM/TSP vs Deteriorating HAM/TSP **Data are expressed as median [range].　 † By Mann-Whitney test ‡By Fisher’s exact test OMDS = Osame’s Motor Disability Score.
